# Behavior Discovery and Alignment of Articulated Object Classes from Unstructured Video

**DOI:** 10.1007/s11263-016-0939-9

**Published:** 2016-08-10

**Authors:** Luca Del Pero, Susanna Ricco, Rahul Sukthankar, Vittorio Ferrari

**Affiliations:** 1 IPAB, School of Informatics, University of Edinburgh, Crichton Street 10, Edinburgh, EH8 9AB UK; 2 Google, 1600 Amphitheatre Pkwy, Mountain View, CA 94043 USA

**Keywords:** Articulated motion, Behavior discovery, Video sequence alignment, Weakly supervised learning from video

## Abstract

We propose an automatic system for organizing the content of a collection of unstructured videos of an articulated object class (e.g., tiger, horse). By exploiting the recurring motion patterns of the class across videos, our system: (1) identifies its characteristic behaviors, and (2) recovers pixel-to-pixel alignments across different instances. Our system can be useful for organizing video collections for indexing and retrieval. Moreover, it can be a platform for learning the appearance or behaviors of object classes from Internet video. Traditional supervised techniques cannot exploit this wealth of data directly, as they require a large amount of time-consuming manual annotations. The behavior discovery stage generates temporal video intervals, each automatically trimmed to one instance of the discovered behavior, clustered by type. It relies on our novel motion representation for articulated motion based on the displacement of ordered pairs of trajectories. The alignment stage aligns hundreds of instances of the class to a great accuracy despite considerable appearance variations (e.g., an adult tiger and a cub). It uses a flexible thin plate spline deformation model that can vary through time. We carefully evaluate each step of our system on a new, fully annotated dataset. On behavior discovery, we outperform the state-of-the-art improved dense trajectory feature descriptor. On spatial alignment, we outperform the popular SIFT Flow algorithm.

## Introduction

**Fig. 1:**
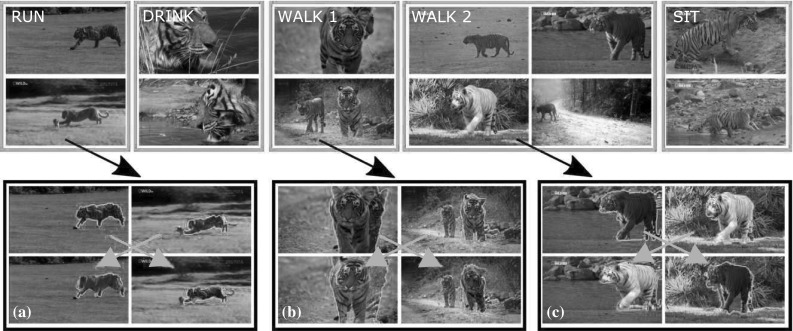
Output of our system. *Top:* examples of the behaviors discovered automatically from a collection of unstructured videos of an object class (*tiger*). From *left* to it right running, drinking, two different types of walking, and sitting down. Our system uses a new descriptor for articulated motion that analyzes the displacement of pairs of trajectories. It is fully automatic: the class label is the only supervision we require. Videos with more behaviors are available at Del Pero et al. (2015b). *Bottom:* within each type of behavior, we find pairs of short sequences where the foreground moves in a consistent manner (*third row*), and align them to a great accuracy (*fourth row*). Here we show an alignment example from the running behavior (**a**), and one each from the two different types of walking (**b**, **c**). The use of motion enables aligning instances despite large variations in appearance (e.g., *white and orange tigers*, *adults* and *cubs*). This step is also fully automatic

Our goal is to automatically organize the content of a collection of unstructured videos of an articulated object class under weak supervision, i.e., only knowing that the object class actually appears in each video (e.g., tiger, Fig. 1). The main contribution of this paper is a fully automatic system that inputs videos of an articulated object class and discovers its characteristic behaviors (e.g., running, walking, sitting down, Fig. 1, top), and also recovers the spatial alignment across different instances of the same behavior (Fig. 1, bottom).

Organizing unstructured video is important for a wide variety of applications, such as video indexing and retrieval (e.g., the TRECVid conference series Smeaton et al. 2006), video database summarization (e.g., Tompkin et al. 2012; Wan and Mérialdo 2009), and computer graphics applications (e.g., replacing an instance in a video with one from a different video, Fig. 1). Moreover, it can help generate training data for supervised systems for action recognition (e.g., Wang and Schmid 2013; Yuan et al. 2009; Schuldt et al. 2004; Gorelick et al. 2007) and object class detection (e.g., Felzenszwalb and Huttenlocher 2005; Bourdev and Malik 2009; Felzenszwalb et al. 2010; Wang et al. 2013; Girshick et al. 2014). These methods cannot fully exploit the abundance of unstructured Internet videos due to the prohibitive cost of generating ground-truth annotations, which explains the recent interest in learning from video under weak supervision (Leistner et al. 2011; Prest et al. 2012; Tang et al. 2013).

Our method requires very little supervision (one class label per video), and could potentially replace the tedious and time-consuming manual annotations needed by supervised recognition systems. For example, action recognition systems are typically trained on clips of human actors performing scripted actions (Yuan et al. 2009; Ryoo and Aggarwal 2009; Schuldt et al. 2004; Gorelick et al. 2007), usually trimmed to contain a single action (Kuehne et al. 2011; Soomro et al. 2012). Discovering the behaviors of a class from unstructured video could replace the process of searching for examples of each behavior, as well as temporally segmenting them out of the videos by hand. Analogously, traditional supervised methods for learning models of object classes from still images (Cootes et al. 1998; Felzenszwalb and Huttenlocher 2005; Bourdev and Malik 2009; Felzenszwalb et al. 2010; Wang et al. 2013; Girshick et al. 2014) do not easily transfer to videos as they require expensive location annotations. The alignments recovered by our method could potentially replace the manual correspondences needed by most popular methods for learning object classes (Dalal and Triggs 2005; Felzenszwalb et al. 2010; Viola et al. 2005; Cinbis et al. 2013; Wang et al. 2013; Girshick et al. 2014), including those requiring part-level annotations (Felzenszwalb and Huttenlocher 2005; Bourdev and Malik 2009; Azizpour and Laptev 2012). They can also enable annotating large collections with little manual effort via knowledge transfer (Vezhnevets and Ferrari 2014; Kuettel et al. 2012; Lampert et al. 2009; Fei-Fei et al. 2007; Malisiewicz et al. 2011). One could provide manual annotations only for a few instances (e.g., segmentation masks Malisiewicz et al. 2011; Vezhnevets and Ferrari 2014; Kuettel et al. 2012 or 3-D models Malisiewicz et al. 2011; Tighe and Lazebnik 2013), and then propagate them automatically to many more instances via the recovered alignments.

Our focus is on highly articulated, deformable objects like animals. Such classes are typically challenging, as they exhibit a much wider variety of interesting behaviors compared to more rigid objects (e.g., a train). Moreover, aligning such objects is challenging due to their deformable nature. These are also the reasons why articulated classes typically require a greater annotation effort than rigid ones (Felzenszwalb and Huttenlocher 2005; Bourdev and Malik 2009; Yang and Ramanan 2013).

A preliminary version of this work appeared at CVPR 2015 (Del Pero et al. 2015a) covering the behavior discovery stage. In this journal paper, we introduce the spatial alignment stage and present a more extensive experimental evaluation.

## Overview of Our Approach

Given unstructured videos of an articulated object class, we discover the class behaviors and recover spatial alignments across different class instances (Fig. 1). We exploit the nature of video and recent advances in motion analysis (Papazoglou and Ferrari 2013; Wang et al. 2011; Wang and Schmid 2013) to make our system fully automatic, for example we use motion segmentation (Papazoglou and Ferrari 2013) to estimate the object’s 2-D location.

To model the motion of an articulated object, we introduce a new descriptor that captures the relative motion of its parts, for example the knee and the ankle of an animal walking. We do this by analyzing the relative displacement of pairs of point trajectories (PoTs). PoTs are a key component of this work, which we discuss and evaluate in detail.

Our system consists of two main stages: behavior discovery and spatial alignment. The behavior discovery builds on the PoTs descriptor. For the spatial alignment, we introduce a technique for aligning short video sequences of the same object class based on thin plate splines (TPSs).

*Pairs of trajectories* (Sect. 3). We model articulated motion by analyzing the relative displacement of large numbers of ordered trajectory pairs (PoTs). The first trajectory in the pair defines a reference frame in which the motion of the second trajectory is measured. We preferentially sample pairs across joints, resulting in features particularly well-suited to representing the behavior of articulated objects. This has greater discriminative power than state-of-the-art features defined using single trajectories in isolation (Wang et al. 2011; Wang and Schmid 2013).

In contrast to other popular descriptors (Jain et al. 2013; Wang et al. 2011; Wang and Schmid 2013), PoTs are defined solely by motion and so are robust to appearance variations within the object class. In cases where appearance proves beneficial for discriminating between behaviors of interest, it is easy to combine PoTs with standard appearance features.

**Fig. 2:**
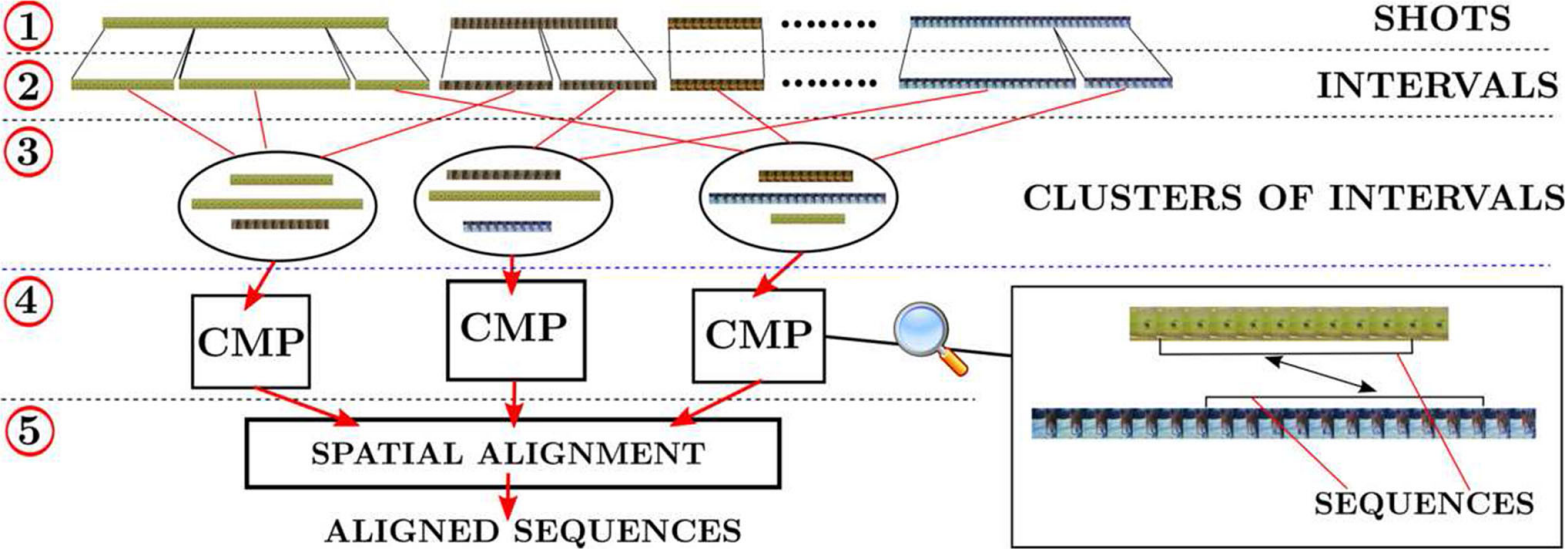
System architecture and terminology (Sect. 2.1). The input is a collection of shots showing the same class (*top*), which can be of any length. We first extract foreground masks (Papazoglou and Ferrari 2013). We then extract PoTs descriptors from each shot (*step 1*, Sect. 3). Each shot is then partitioned into shorter temporal intervals that are each likely to contain a single behavior (*step 2*, Sect. 4.1), which we cluster using PoTs (*step 3*, Sect. 4.2). For each two intervals in the same cluster, we extract pairs of short sequences showing consistent foreground motion (CMPs), which become candidates for spatial alignment (*step 4*, Sect.5.1). Last, we align the two sequences of each CMP (*step 5*, Sects. 5.2, 5.3)

*Behavior discovery (Sect.* 4). Our method does not require knowledge of the number or types of behaviors, nor that instances of different behaviors be temporally segmented within a video. Instead, we leverage that behaviors exhibit consistency across videos, resulting in characteristic motion patterns. Our method identifies motion patterns that recur across several videos: it temporally segments them out of the input videos, and clusters them by type. For this, we use PoTs as motion representation, which allow us to distinguish between fine-grained behaviors, such as walking and running. Note that our *unsupervised* discovery is very different from simply classifying fixed temporal chunks of video into behaviors (e.g., action recognition in UCF-101 Soomro et al. 2012), which requires *supervision* (e.g., training data for each behavior), and does not need to address the temporal segmentation.

*Spatial alignment (Sect.* 5). Consider the problem of aligning any two instances of a tiger. This is challenging due to differences in viewpoint (e.g., frontal and side), pose (jumping and sitting down), and appearance (cub and adult). The behavior discovery stage simplifies the problem by forming clusters of videos exhibiting a consistent set of poses (e.g., walking, jumping). However, aligning two individual frames with traditional techniques for aligning still images (Barnes et al. 2010; Liu et al. 2008; Hartley and Zisserman 2000; Lowe 2004) typically fails even in this scenario, due to the significant appearance variations across instances and pose variations within the same behavior (e.g., different phases of walking). Instead, we align two short temporal sequences where the objects exhibit consistent motion (we identify these sequences automatically within the behavior clusters).

We exploit the consistency in object motion to establish reliable point correspondences between the sequences, and combine this with edge features to align them with great accuracy (Fig. 1). We model the transformation between the two sequences using a series of TPSs (Wahba 1990). TPS are an expressive non-rigid mapping that can accommodate for the deformations of complex articulated objects. TPS have been used before mostly for registration (Chui and Rangarajan 2003) and shape matching (Ferrari et al. 2010) in still images. We extend these ideas to video by fitting TPS that vary smoothly in time.

### System Architecture

We provide here a high-level description of the architecture of our system (Fig. 2).

*Input video shots.* The input is a collection of *video shots* of the same object class. By shot we mean a sequence of frames without scene transitions (Kim and Kim 2009). We work with Internet videos automatically partitioned into shots by thresholding histogram differences across consecutive frames (Prest et al. 2012; Kim and Kim 2009). The only supervision given is the knowledge that each shot contains the object class.

*Foreground masks.* We use the fast video segmentation technique (Papazoglou and Ferrari 2013) on each input shot, to automatically segment the foreground object from the background. These foreground masks remove features on the background and facilitate the entire process. To handle shots containing multiple moving objects, we only keep the largest connected component in the foreground mask. This typically corresponds to the largest object in the shot (a similar strategy is used in Papazoglou and Ferrari 2013 for evaluation).

*Step 1: PoT extraction (Sect.* 3). We extract PoTs from each input shot, which we use as features in the following steps.

*Step 2: Partitioning into temporal intervals (Sect.* 4.1). Clustering the input shots directly would fail to discover behaviors, since each shot typically contains several different behaviors. For example, a tiger may walk for a while, then sit down and finally stretch. We use motion cues to partition shots into *single-behavior intervals*, e.g., a “walking”, a “sitting down” and a “stretching” interval.

*Step 3: Behavior discovery by clustering (Sect.* 4.2). We use the extracted PoTs to build a descriptor for each interval from step 2, and cluster them. At this stage of the pipeline, each cluster contains several intervals of the same behavior, each temporally trimmed to its duration.

*Step 4: Candidates for spatial alignment (Sect.* 5.1). We exploit the consistent motion of two intervals in the same behavior cluster to drive their alignment. However, we cannot expect the motion to be consistent for their entire duration: this would require that the object performs exactly the same movements in the same order in both intervals. Hence, we identify a few shorter sequences of fixed length between the two intervals that exhibit consistent foreground motion. We term these *consistent motion pairs* (CMPs), and use them as candidates for spatial alignment.

**Fig. 3:**
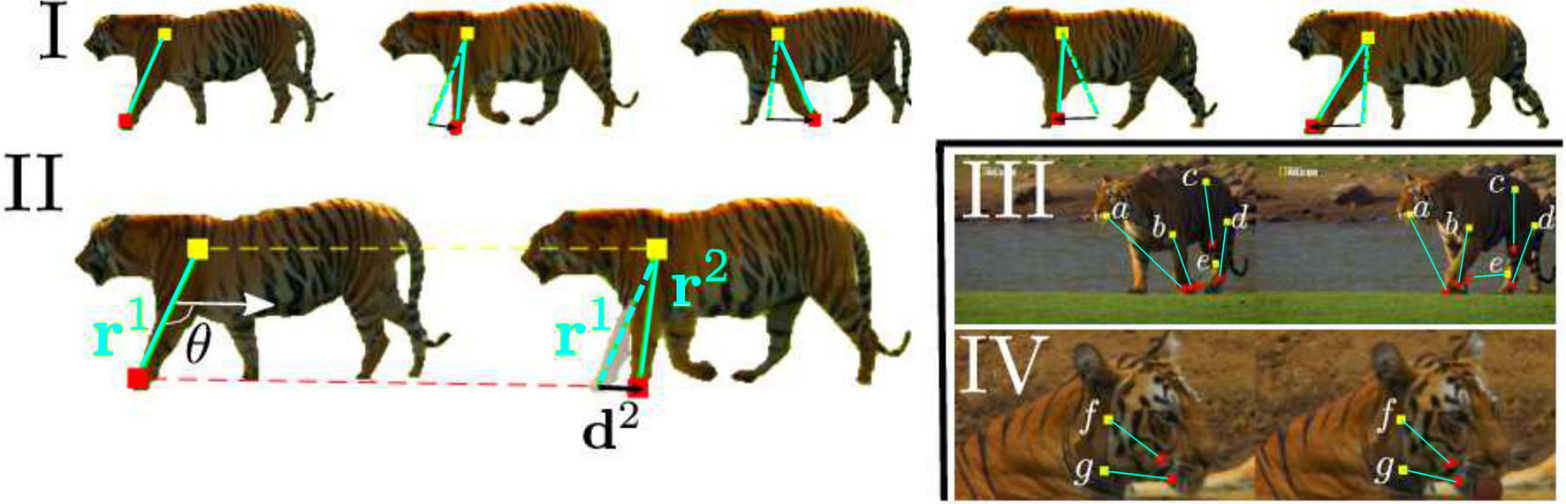
Modeling articulated motion with PoTs. Two trajectories in a PoT are ordered based on their deviation from the median velocity of the object: the anchor (*yellow*) deviates less than the swing (*red*). In **I**, the displacement of the swing relative to the anchor follows the swinging motion of the paw with respect to the shoulder. While both move forward as the *tiger* walks, the *paw* is actually moving backwards in a *coordinate* system *centered* at the *shoulder*. This back-and-forth motion is captured by the relative displacement vectors of the pair (in *black*) but missed when individual trajectories are used alone. The PoT descriptor is constructed from the angle $\theta$ and the *black* vectors $\mathbf {d^k},$ shown in **II** (Sect. 3.1). The two trajectories in a PoT are selected such that they track object parts that move differently. A few selected PoTs are shown in **III** and **IV**. *Paws* move differently than the *head* (*a*), *hip* (*c*), *knees* (*b*, *d*), or other *paws* (*e*). In **IV**, the *head* rotates relative to the neck, resulting in different PoTs (*f*, *g*). Our method selects these PoTs without requiring prior knowledge of the object topology (Sect. 3.2) (Color figure online)

**Fig. 4:**
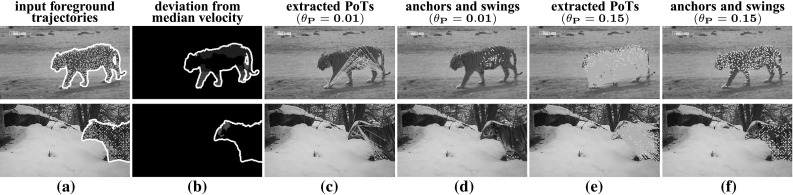
PoT selection on two different examples: a *tiger* walking (*top*) and one turning its *head* (*bottom*). We construct PoT candidates from the trajectories on the foreground mask (**a**), using all possible pairs (Sect. 3.2). We prefer candidates where the anchor is closer to the median foreground velocity, denoted by *dark areas* in **b**, while the swing follows a different motion (*bright areas*). We keep the highest $\theta _{P} \%$ ranking candidates according to this criterion. We show the selected PoTs for two different values of $\theta _{P}$ (**c**, **e**). Too strict a $\theta _{P}$ ignores many interesting PoTs (**c**), like those involving trajectories on the *head* in the *top row*. We also show the trajectories used as anchors (*yellow*) and swings (*red*) without the lines connecting them (**d**, **f**). Imagine connecting any anchor with any swing: in most cases, the two follow different, independently moving parts of the object, which is the key requirement of a PoT. We use $\theta _{P} = 0.15$ in our experiments (**e**, **f**) (Color figure online)

*Step 5: Spatial alignment (Sects.* 5.2, 5.3). For each CMP, we attempt to align its two sequences. If the algorithm succeeds, we output the aligned CMP. We consider two different spatial alignment models: homographies and TPS.

### Experiments Overview

We present extensive quantitative evaluation on a new dataset containing several hundreds videos of three articulated object classes (dogs, horses and tigers, Sect. 7.1). We produced the annotations necessary to evaluate the two outputs of our method: (1) per-frame behavior labels in over 110,000 frames to evaluate behavior discovery, and (2) 2-D positions of 19 landmarks (e.g., left eye, front right ankle) in over 35,000 frames to evaluate spatial alignment.

The results demonstrate that our method can discover behaviors from a collection of unconstrained video, while also segmenting out behavior instances from the input videos (Sects. 7.2, 7.3). On these tasks, PoTs perform significantly better than existing appearance- and trajectory-based descriptors (e.g., histogram of oriented gradient (HOG) and dense trajectory features, DTFs Wang and Schmid 2013).

Our TPS based alignment outperforms existing alternatives that are either unsuitable for articulated objects (e.g., homographies Caspi et al. 2006; Hartley and Zisserman 2000; Lowe 2004), or designed to align still images (e.g., the popular SIFT Flow algorithm Liu et al. 2008). Our system recovers approximately 1000 pairs of correctly aligned sequences from 100 real-world video shots of tigers, and 800 aligned sequences from 100 shots of horses. As the recovered alignment is between *sequences*, this entails correspondences between several thousand pairs of frames (Sect. 7.4.3).

## Pair of Trajectories (PoTs)

We represent articulated object motion using a collection of ordered PoTs, tracked over *n* frames. We compose PoTs from the trajectories extracted with a dense point tracker (e.g., Wang and Schmid 2013): only two trajectories following parts of the object moving relatively to each other are selected as a PoT, as these are the pairs that move in a consistent and distinctive manner across different instances of a specific behavior. For example, the motion of a pair connecting a tiger’s knee to its paw consistently recurs across videos of walking tigers (Figs. 3, 4). By contrast, a pair connecting two points on the chest (a rather rigid structure) may be insufficiently distinctive, while one connecting the tip of the tail to the nose may lack consistency. Note also that a trajectory may simultaneously contribute to multiple PoTs (e.g., a trajectory on the front paw may form pairs with trajectories from the shoulder, hip, and nose).

Although we often refer to PoTs using semantic labels for the location of their component trajectories (eye, shoulder, hip, etc.), these are used only for convenience. PoTs do not require semantic understanding or any part-based or skeletal model of the object, nor are they specific to a certain object class. Furthermore, the collection of PoTs is more expressive than a simple star-like model in which the motion of point trajectories are measured relative to the center of mass of the object (i.e., normalizing by the dominant object motion). For example, we find the “walking” cluster (Fig. 1) based on PoTs formed by various combinations of head–paw (Fig. 3 III, a), hip–knee (c), knee–paw (b, d), or even paw–paw trajectories (e).

Figure 3 (III, IV) shows a few examples of PoTs selected from two tiger videos. We define PoTs in Sect. 3.1, while we explain how to select PoTs from real videos in Sect. 3.2.

### PoT Definition

*PoT ordering: anchors and swings.* The two trajectories in a PoT are *ordered*, i.e., we always measure the displacement of the second trajectory (*swing*) in the local coordinate frame defined by the first (*anchor*). We select as anchor the trajectory whose velocity is closer to the median velocity of pixels on the foreground mask, aggregated over the length of the PoT (Sect. 3.2). This approximates the median velocity of the whole object. This criterion generates a stable ordering, repeatable across the broad range of videos we examine. For example, the trajectories on the legs in Fig. 3 are consistently chosen as swings while those on the torso as anchors.

*Displacement vectors*. In each frame $f_k,$ we compute the vector $\mathbf {r^{k}}$ from anchor to swing (cyan lines in Fig. 3). Starting from the second frame, a displacement vector $\mathbf {d^{k}}$ is computed by subtracting the vector $\mathbf {r^{k-1}}$ of the previous frame (dashed cyan) from the current $\mathbf {r^{k}}$ (solid cyan). $\mathbf {d^k}$ captures the motion of the swing relative to the anchor by canceling out the motion of the latter. Naively employing the cyan vectors $\mathbf {r^k}$ as raw features does not capture relative motion as effectively, because the variation in $\mathbf {r^k}$ through time is dominated by the spatial arrangement of anchor and swing rather than by the change in relative position between frames (compare the magnitudes of the cyan and black vectors in Fig. 3). Note this way of computing the displacement vectors is invariant to camera panning, since the relative motion of the trajectories does not change whether the camera is static or panning.

*PoT descriptor*. The descriptor *P* has two parts: (1) the initial position of the swing relative to the anchor, and (2) the sequence of normalized displacement vectors over time:

1 $$\begin{aligned} P=\left( \theta ,\, \frac{\mathbf {d^2}}{D}, \ldots , \frac{\mathbf {d^n}}{D} \right) , \end{aligned}$$

where $\theta$ is the angle from anchor to swing in the first frame (in radians) and the normalization factor is the total displacement $D=\sum _{k=2}^{n}||\mathbf {d^{k}}||.$ The DTFs descriptor (Wang et al. 2011) employs a similar normalization. Note also that the first term in *P* records only the angle (and not the magnitude) between anchor and swing; this retains scale invariance and enables matching PoTs between objects of different size. The dimensionality of *P* is $2\cdot (n-1)+1;$ in all of our experiments $n = 10.$

### PoT Selection

We explain here how to automatically form PoTs out of a set of trajectories extracted with a dense point tracker (Wang and Schmid 2013). We start with a summary of the process and give more details later. First, we remove trajectories on the background using the foreground masks. Then, for each frame *f* we build the set $\mathcal {P}_{f}$ of PoTs starting at that frame. For computational efficiency, we directly set $\mathcal {P}_{f}=\emptyset$ for any frame unlikely to contain articulated motion. Otherwise, we form candidate PoTs from all pairs of foreground trajectories $\{t_i,\,t_j\}$ extending for at least *n* frames after *f*. Finally, we retain in $\mathcal {P}_{f}$ the candidates most likely to be on object parts moving relative to each other.

*Removing background trajectories*. State-of-the-art point trajectories already attempt to limit trajectories to foreground objects (Wang and Schmid 2013), but often fail on the wide range of videos we use. The video segmentation technique we use (Papazoglou and Ferrari 2013) handles unconstrained video, and reliably detects articulated objects even under significant motion and against cluttered backgrounds. Hence, we remove point trajectories that fall outside the foreground mask produced by Papazoglou and Ferrari (2013). Results show that our overall method is robust to inaccurate foreground masks because they only affect a fraction of the PoT collection (Sect. 7.2).

We also use the masks to estimate the median velocity of the object, computed as the median optical flow displacement over all pixels in the mask.

*Pruning frames without articulated motion*. A frame is unlikely to contain articulated motion (hence PoTs) if the optical flow displacement of foreground pixels is uniform. This happens when the entire scene is static, or the object moves with respect to the camera but the motion is not articulated. We define $s(f) = \frac{1}{n}\sum _{i=f}^{f+n-1}\sigma _{i},$ where $\sigma _{i}$ is the standard deviation in the optical flow displacement over the foreground pixels at frame *i* normalized by the mean, and *n* the length of the PoT. We set $\mathcal {P}_{f}=\emptyset$ for all frames where $s(f) < \theta _{F},$ thereby pruning frames unlikely to contain any PoT. We choose $\theta$ on 16 cat videos in which we manually labeled frames without articulated motion. We set $\theta _{F}=0.1,$ which yields precision 0.95 and recall 0.75 (very similar performance is achieved for $0.05 \le \theta _{F} \le 0.2$).

*PoT candidates and selection*. The candidate PoTs for a frame *f* are all ordered PoTs $\{t_i,\,t_j\}$ that start in *f* and exist in the following $n-1$ frames (Fig. 4a). We score a candidate pair $\{t_i,\,t_j\}$ using

2 $$\begin{aligned} \mathrm {S}\left( \left\{ t_i=a,\, t_j=s\right\} \right) = \sum _{k=f}^{f+n-1}\left\| v_{s}^{k} - v_{m}^{k}\right\| - \left\| v_{a}^{k} - v_{m} ^{k}\right\| , \nonumber \\ \end{aligned}$$

where $v_{m}^{k}$ is the median velocity at frame *k*,  and $v_{s}^{k},\,v_{a}^{k}$ are the velocities of the swing and anchor. The first term favors pairs where the swing velocity deviates a lot from the median, while the second term favors pairs where the anchor velocity is close to the median. As seen in Fig. 4, this generates a stable PoT ordering, for example the swings fall on the legs as the tiger walks (top), or on the turning head (bottom). We rank all candidates using (2) and retain the top $\theta _{P} \%$ candidates as PoTs $\mathcal {P}_{f}$ for this frame (Fig. 4c–f). In all experiments we use $\theta _{P} = 0.15.$ Since we score all possible pairs with (2), a particular trajectory can serve as anchor in one pair and as swing in a different pair, depending on the velocity of the other trajectory in the pair.

## Behavior Discovery

The behavior discovery stage inputs a set of shots $\mathcal {S}$ of the same class (Fig. 2, top) and outputs clusters of temporal intervals, $\mathcal {C}=(c_{1},\ldots ,c_{k})$ corresponding to behaviors (step 3 in Fig. 2). For the “tiger” class, we would like a cluster with tigers walking, one with tigers turning their head, and so on. We first temporally partition shots into single behavior intervals (Sect. 4.1). Then we cluster these intervals to discover recurring behaviors (Sect.4.2).

### Temporal Partitioning

An input shot typically contains several instances of different behaviors each. It would be easier to cluster intervals corresponding to just one instance of a behavior, and ideally covering its whole duration. Here we partition each shot into such single behavior intervals. Boundaries between such intervals cannot be detected using simple color histogram differences (unlike shot boundaries Kim and Kim 2009). Further, naively partitioning into fixed-length intervals invariably ends up either over- or under-partitioning. Instead, we use an adaptive strategy based on two different motion cues: pauses and periodicity.

*Partitioning on pauses.* The object often stays still for a brief moment between two different behaviors. We detect such pauses as sequences of three or more frames without articulated object motion (Sect. 3.2).

*Partitioning based on periodicity.* As some sequences lack pauses between different, but related behaviors (e.g., from walking to running), we also partition based on periodic motion. For this we use time–frequency analysis, as periodic motion patterns like walking, running, or licking typically generate peaks in the frequency domain (examples available on our website Del Pero et al. 2015b).

We model an interval as a time sequence $s(t)=b_{f^{t}}^{P},$ where $b_{f^{t}}^{P}$ is a bag-of-words (BoWs) of PoTs at frame $f^{t}.$ We convert *s*(*t*) to *V* one-dimensional sequences and sum the fast Fourier transform (FFT) of the individual sequences in the frequency domain (*V* is the codebook size). If the height of the highest peak is ${\ge } \theta _{H},$ we consider the interval as periodic. We normalize the total energy to make sure it integrates to 1. Using the sum of the FFTs makes the approach more robust, since peaks arise only if several codewords recur with the same frequency.

Naively doing time–frequency analysis on an entire interval typically fails because it might contain both periodic and non-periodic motion (e.g., a tiger walks for a while and then sits down). Hence, we consider all possible sub-intervals using a temporal sliding window and label the one with the highest peak as periodic, provided its height ${\ge } \theta _{H}.$ The remaining segments are reprocessed to extract motion patterns with different periods (e.g., walking versus running) until no significant peaks remain. For robustness, we only consider sub-intervals where the period is at least five frames and the frequency at least three (i.e., the period repeats at least three times). We empirically set $\theta _{H} = 0.1,$ which produces very few false positives.

### Clustering Intervals

We use *k*-means to form a codebook from one million PoT descriptors randomly sampled from all intervals, using Euclidean distance.^1^ We run *k*-means eight times and choose the clustering with lowest energy to reduce the effects of random initialization (Wang and Schmid 2013). We then represent an interval as a BoW histogram of the PoTs it contains (L1-normalized).

We cluster the intervals using hierarchical clustering with complete-linkage (Johnson 1967). We found this to perform better than other clustering methods (e.g., single-linkage, *k*-means) for both PoTs and the improved DTFs, (IDTFs; Wang and Schmid 2013) descriptor, which we compare against in the experiments (Sect. 7.2).

Hierarchical clustering requires computing distances between items. Given BoWs of PoTs $b_{u}$ and $b_{v}$ for intervals $I_{u}$ and $I_{v},$ we use

3 $$\begin{aligned} \mathrm {d}\left( I_{u},\,I_{v}\right) = {-}\mathrm {exp}\left( -\left( 1-\mathrm {HI}\left( b_{u},\,b_{v}\right) \right) \right) , \end{aligned}$$

where $\mathrm {HI}$ denotes histogram intersection. We found this to perform slightly better than the $\chi ^2$ distance. Note that this function can be also used on BoWs of descriptors other than PoTs. Additionally, it can be extended to handle different descriptors that use multiple feature channels, such as IDTFs (Wang and Schmid 2013). In this case, the interval representation is a set of BoWs $(b_{u}^{1},\ldots ,b_{u}^{C}),$ one for each of the *C* channels. Following Wang and Schmid (2013), we combine all channels into a single distance function

4 $$\begin{aligned} \mathrm {d}\left( I_{u},\,I_{v}\right) = {-}\mathrm {exp}\left( -\sum _{i=1}^{C} \frac{1-\mathrm {HI}(b_{u}^{i},\,b_{v}^{i})}{A_{i}}\right) , \end{aligned}$$

where $A_{i}$ is the average value of $(1-\mathrm {HI})$ for channel *i*.

## Sequence Alignment

Having clustered intervals by behavior type, we can search for suitable candidates for spatial alignment, i.e., pairs of short sequences with consistent foreground motion (dubbed CMPs, Fig. 2, step 4). This is discussed in Sect. 5.1.

We have explored a variety of approaches for sequence alignment, and report on two representative methods here (Fig. 6). The first is a coarse, global alignment generated by fitting a single homography to foreground trajectory descriptors matched between the two sequences (Sect. 5.2). The second approach fits a finer, non-rigid TPS mapping to edge points extracted from the foreground regions of each frame. We allow TPS to vary smoothly through the sequence (Sect. 5.3). As we show in our experiments, the TPS prove more suitable for aligning complex articulated objects (Sect. 7.4).

### Extracting CMP Candidates

**Fig. 5:**
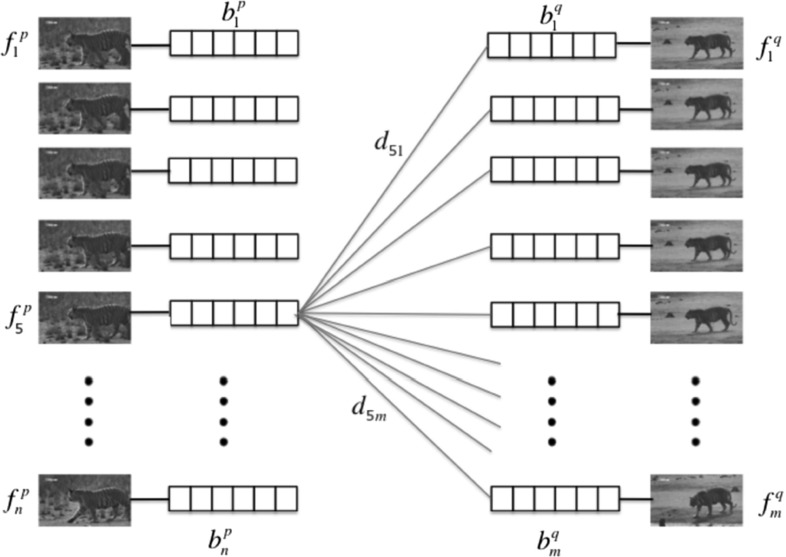
Extracting CMPs from two intervals. First, we approximate the pairwise distance between frames as the *histogram* distance between their BoWs (which contains all motion descriptors through the frame, Sect. 5.1). Then we keep as CMPs the top scoring pairs of sequences of length *T* with respect to (5). For the intervals above, the number of pairs of sequences to score is $(n-T)\cdot (m-T)$

Given two intervals *p* and *q* in the same behavior cluster, we extract as CMPs the top 10 ranked pairs of subsequences between them according to the following metric (Fig.5). Let $d_{ij}$ be the HI between BoW descriptors computed for frame *i* in *p* and frame *j* in *q*. We compute $d_{ij}$ just like in (4), except that we aggregate only the descriptors in the specific frame rather than the whole interval. The similarity between the *T*-frame subsequence of *p* starting at frame *i* and the subsequence of *q* starting at frame *j* is

5 $$\begin{aligned} {\mathrm {s}\left( \left[ f_{i}^{p},\ldots ,f_{i+T-1}^{p}\right] , \left[ f_{j}^{q},\ldots ,f_{j+T-1}^{q}\right] \right) =\sum _{t=0}^{T-1}d_{(i+t)(j+t)}}. \end{aligned}$$

This measure preserves the temporal order of the frames, whereas aggregating the BoW over the whole sequences as in (4) would not. To compute $d_{ij}$ we combine two channels: PoTs and motion boundary histogram (MBH; Wang and Schmid 2013).

**Fig. 6:**
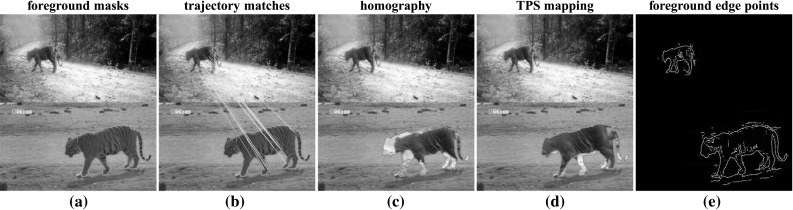
Aligning sequences with similar foreground motion. We first estimate a foreground mask (*green*) using motion segmentation (**a**). We then fit a homography to matches between point trajectories (**b**, Sect. 5.2). In **c** we project the foreground pixels in the first sequence (*top*) onto the second (*bottom*) with the recovered homography. This global, coarse mapping is often not accurate (note the misaligned *legs* and *head*). We refine it by fitting thin-plate splines (TPSs) to edge points extracted from the foreground (**e**, Sect. 5.3). The TPS mapping is non-rigid and provides a more accurate alignment for complex articulated objects (**d**) (Color figure online)

We found this scheme extracts CMPs that reliably show similar foreground motion and form good candidates for spatial alignment (Sect. 7.4.2). Restricting the search of CMPs within a behavior cluster prunes unsuitable candidates (e.g., a tiger jumping and one rolling on the ground). Using only the top 10 pairs according to (5) further reduces the search space, extracting a manageable set of CMPs (e.g., 3000 CMPs in a dataset of 100 tiger shots, where we have to align 300 pairs of intervals after the behavior discovery stage, Sect. 7.4.3). The alternative strategy of trying to align all possible pairs of subsequences in the input shots is instead quadratic in the number of input frames (${\sim }300$ million), and thus computationally impractical.

### Homography-Based Sequence Alignment

Traditionally, homographies are used to model the mapping between two still images, and are estimated from a set of 2-D point correspondences (Hartley and Zisserman 2000). Instead, we estimate the homography from trajectory correspondences between two sequences (in a CMP). We first review the traditional approach (Sect. 5.2.1), and then present our extensions (Sects. 5.2.2–5.2.3).

#### Homography Between Still Images

A 2-D homography $H_{uv}$ is a $3\times 3$ matrix that can be determined from four or more point correspondences $X_{u} \leftrightarrow X_{v}$ by solving

6 $$\begin{aligned} X_u = H_{uv}X_v. \end{aligned}$$

RANSAC (Fischler and Bolles 1981) estimates a homography from a set of putative correspondences $\mathcal {P}_{uv} = \{(x_u,\,y_u) \leftrightarrow (x_v,\,y_v)\}$ that may include outliers. Traditionally, $\mathcal {P}_{uv}$ contains matches between local appearance descriptors, like SIFT (Lowe 2004). RANSAC operates by running a large number of trials, each consisting of randomly sampling four point correspondences from $\mathcal {P}_{uv},$ fitting a homography to them, and counting the number of inliers it has in the whole set $\mathcal {P}_{uv}.$ In the end, RANSAC returns the homography with the largest number of inliers.

#### Homography Between Video Sequences

**Fig. 7:**
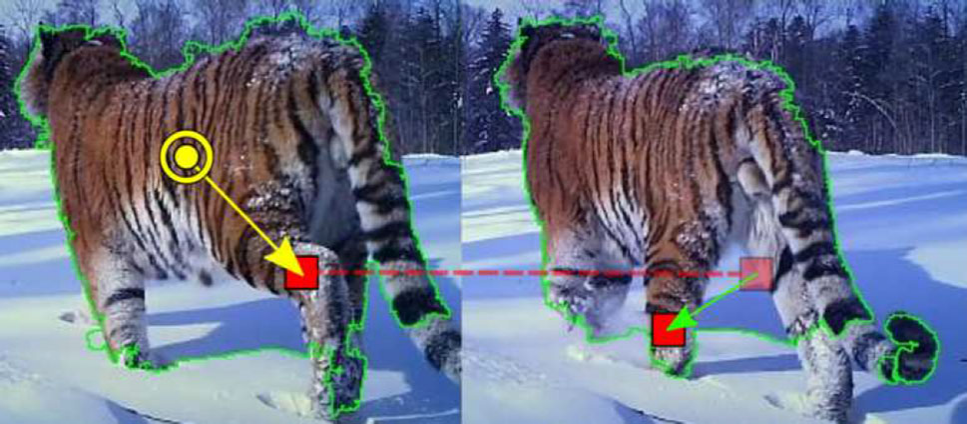
Modifying the TS descriptor. The TS descriptor is the concatenation of the 2-D displacement vectors (*green*) of a trajectory across consecutive frames. TS works well when aggregated in unordered representations like bag-of-words (Wang and Schmid 2013), but matches found between individual trajectories are not very robust, e.g., the TS descriptors for the trajectories on the *torso* of a *tiger* walking are almost identical. We make TS more discriminative by appending the vector (*yellow*) between the trajectory and the center of mass of the foreground mask (*green*) in the frame where the trajectory starts (Sect. 5.2.2). We normalize this vector by the *diagonal* of the bounding *box* of the foreground mask to preserve scale invariance (Color figure online)

In video sequences, we use point trajectories as units for matching, instead of points in individual frames (Fig. 6b). We extract trajectories in each sequence and match them using a modified trajectory shape (TS) descriptor (Wang and Schmid 2013) (Fig. 7). We match each trajectory in the first sequence to its nearest neighbor in the second with respect to Euclidean distance. We use trajectories which are $T=10$ frames long, and only match those starting in the same frame in both sequences. Each trajectory match provides *T* point correspondences (one per frame).

We consider two alternative ways to fit a homography to the trajectory matches, called ‘Independent Matching’ (IM) and ‘Temporal Matching’ (TM). IM treats the point correspondences generated by a single trajectory match independently during RANSAC. TM instead samples four *trajectory* matches at each RANSAC iteration, and solves (6) in the least squares sense using the $4\cdot T$ point correspondences. A trajectory match is considered an outlier only if more than half of its point correspondences are outliers. TM encourages geometric consistency over the duration of the CMP, while IM could potentially overfit to point correspondences in just a few frames. In practice, our experiments show that TM is superior to IM (Sect. 7.4).

We also considered matching PoTs across the sequences instead of individual trajectories, but this is less efficient because each trajectory can be part of many PoTs (we can build $O(n^2)$ PoTs out of *n* trajectories). Computationally, matching two sets of trajectories of size *n* and *m* is *O*(*nm*),  while with PoTs it would be $O(n^2m^2).$

#### Using the Foreground Mask as a Regularizer

**Fig. 8:**
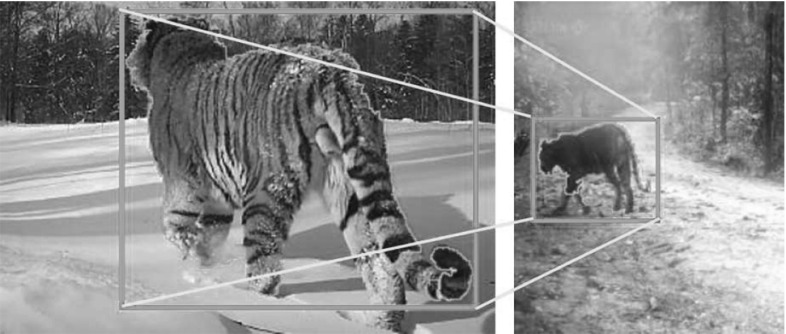
Matching the corners between the bounding *boxes* of the foreground mask provides additional point correspondences (Sect. 5.2.3). These are too coarse to provide a detailed spatial alignment between the two sequences and are also sensitive to errors in the foreground masks, but they are useful when combined with *point* correspondences from trajectory matches (Fig. 9)

**Fig. 9:**
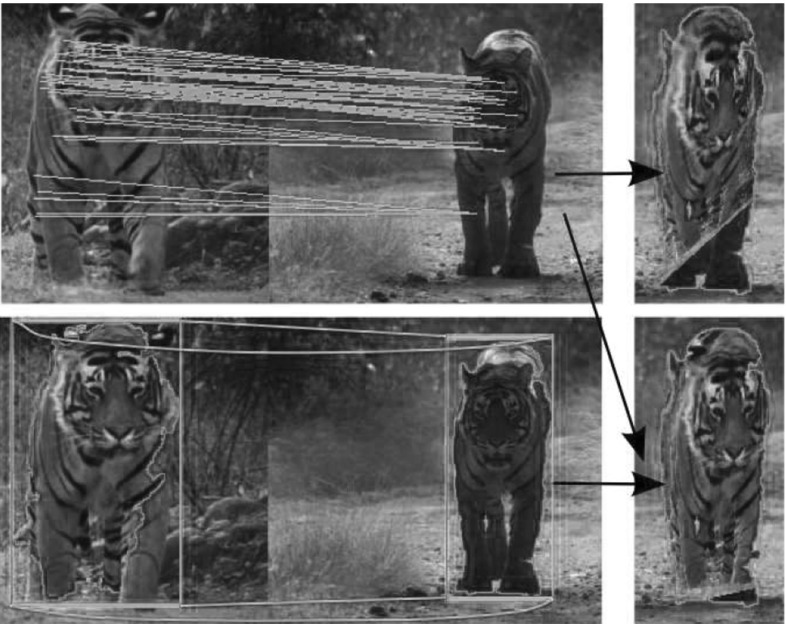
*Top: trajectory* matches (*yellow*) often cover only part of the object. Here, the homography overfits to the correspondences on the *head*, providing an incorrect mapping for the *legs* (*right*). *Bottom:* adding correspondences from the bounding *boxes* of the foreground masks (Papazoglou and Ferrari 2013) provides a more stable mapping (*right*, Sect. 5.2.3). Note how also these correspondences are found automatically by our method (no manual intervention needed) (Color figure online)

The estimated homography tends to be inaccurate when the input matches do not cover the entire foreground (Fig. 9). To address this issue, we note that the bounding boxes of the foreground masks (Papazoglou and Ferrari 2013) induce a very coarse global mapping (Fig. 8). Specifically, we include the correspondences between the bounding box corners $F_{u} \leftrightarrow F_{v}$ in (6):

7 $$\begin{aligned} \min _{H_{uv}} \left\| H_{uv}X_{v}- X_{u}\right\| + \left\| H_{uv}F_{v}- F_{u}\right\| . \end{aligned}$$

This form of regularization makes our method much more stable (Fig. 9).

### Temporal TPS for Sequence Alignment

We now present our approach to sequence alignment based on time-varying TPSs (TTPSs). Unlike a homography, TTPS allows for local warping, which is more suitable for putting different object instances in correspondence. We build on the popular TPS robust point matching algorithm (TPS-RPM; Chui and Rangarajan 2003), originally developed to align point sets between two still images (Sect. 5.3.1). We extend TPS-RPM to align two sequences of frames with a TPS that evolves smoothly over time (Sect. 5.3.2).

#### TPS-RPM

A TPS $\mathrm {f}$ comprises an affine transformation *d* and a non-rigid warp *w*. The mapping is a single closed-form function for the entire space, with a smoothness term $\mathrm {L}(\mathrm {f})$ defined as the sum of the squares of the second derivatives of $\mathrm {f}$ over the space (Chui and Rangarajan 2003). Given two sets of points $\mathcal {U} = \{u_i\}$ and $\mathcal {V} = \{v_i\}$ in correspondence, $\mathrm {f}$ can be estimated by minimizing

8 $$\begin{aligned} \mathrm {E}(\mathrm {f}) = \sum _{i} \left\| u_{i} - \mathrm {f}\left( v_{i}\right) \right\| ^{2} + \lambda \Vert \mathrm {L}(\mathrm {f})\Vert . \end{aligned}$$

$\mathcal {U}$ and $\mathcal {V}$ are typically the position of detected image features (we use edge points, Sect. 5.3.2).

As the point correspondences are typically not known beforehand, TPS-RPM jointly estimates $\mathrm {f}$ and a soft-assign correspondence matrix $M=\{m_{ij}\}$ by minimizing

9 $$\begin{aligned} \mathrm {E}(M,\, \mathrm {f}) = \sum _{i} \sum _{j} m_{ij}\left\| u_{i} - \mathrm {f}\left( v_{j}\right) \right\| ^{2} + \lambda \Vert \mathrm {L}(\mathrm {f})\Vert . \end{aligned}$$

TPS-RPM alternates between updating $\mathrm {f}$ by keeping *M* fixed, and the converse. *M* is continuous-valued, allowing the algorithm to evolve through a continuous correspondence space, rather than jumping around in the space of binary matrices (hard correspondence). It is updated by setting $m_{ij}$ as a function of the distance between $u_{i}$ and $\mathrm {f}(v_{j})$ (Chui and Rangarajan 2003). The TPS is updated by fitting $\mathrm {f}$ between $\mathcal {V}$ and the current estimates $\mathcal {Y}$ of the corresponding points, computed from $\mathcal {U}$ and *M*.

TPS-RPM optimizes (9) in a deterministic annealing framework, which enables finding a good solution even when starting from a relatively poor initialization. The method is also robust to outliers in $\mathcal {U}$ and $\mathcal {V}$ (Chui and Rangarajan 2003).

**Fig. 10:**
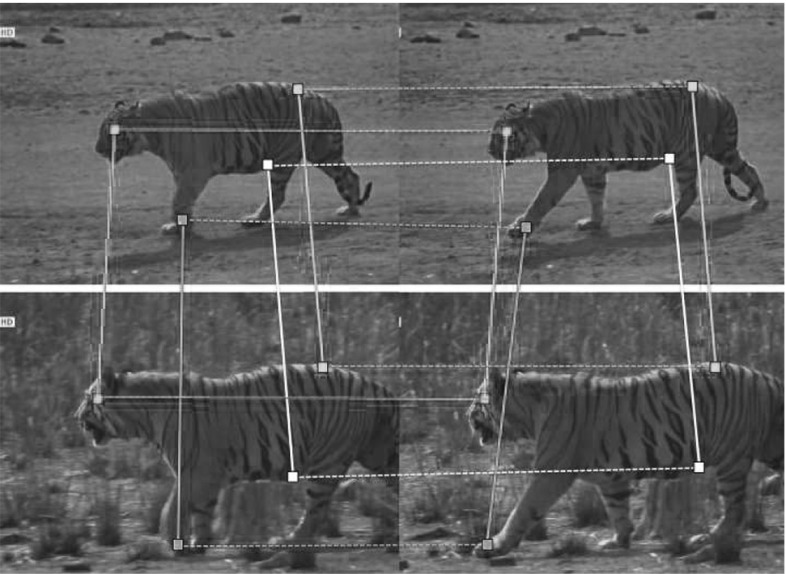
Edge propagation using optical flow. In each sequence, we propagate edge *points* extracted at time *t* using optical flow, independently in each sequence (*dashed lines*). Our TTPS model (Sect. 5.3.2) enforces that the correspondences between edge *points* at time *t* (*solid lines*) be consistent with their propagated version at time $t+1$

#### Temporal TPS

Our goal is to find a series of TPS mappings $\mathcal {F}=\{\mathrm {f}^{1},\ldots ,\mathrm {f}^{T} \},$ one at each frame in the input sequences. We enforce temporal smoothness by constraining each mapping to use a set of point correspondences consistent over time. Let $\mathcal {U}^t = \{u_i^t\}$ be the set of points for frame *t* in the first sequence (with $\mathcal {V}^t$ defined analogously for the second sequence). $\mathcal {U}^t$ contains both edge points extracted in *t* as well as edge points extracted in other frames and propagated to *t* via optical flow (Fig. 10). Each $\mathcal {U}^t$ stores points in the same order, so that $u_i^{1}$ and $u_i^{\tau }\forall \tau > 1$ are related by flow propagation.^2^ We solve for the TTPS $\mathcal {F}$ by minimizing

10 $$\begin{aligned}&\mathrm {E}(\mathcal {M},\, \mathcal {F})\nonumber \\&\quad = \sum _{t} \left( \sum _{i} \sum _{j} m_{ij}^t\left\| u_{i}^{t} - \mathrm {f}^{t}\left( v_{j}^{t}\right) \right\| ^{2} + \lambda \left\| \mathrm {L}\left( \mathrm {f}^{t}\right) \right\| \right) ,\nonumber \\ \end{aligned}$$

subject to the constraint that $m_{ij}^1 = m_{ij}^{\tau }\,\forall i,\,j,\,\tau > 1.$ That is, if two points are in correspondence in frame *t*,  they must still be in correspondence after being propagated to frame $\tau .$

*Inference*. Minimizing (10) is very challenging. In practice, we find an approximate solution by first using TPS-RPM to fit a TPS $\mathrm {f}^\tau$ to the edge points extracted at time $\tau$ only. This is initialized with the homography found in Sect. 5.2.3. Given the constraints on the $m_{ij}^t,\,\mathrm {f}^{\tau }$ fixes the correspondences between $\mathcal {U}^t$ and $\mathcal {V}^t$ in all other frames. We then fit the $\mathrm {f}^{t}\,\forall t\ne \tau$ to these correspondences. We repeat this process starting in each frame (i.e., we try all $\tau \in [1,\ldots ,T]$), generating a total of *T* TTPS candidates. Finally, we return the one with the lowest energy (10). Thanks to this efficient approximate inference, we can apply TTPS to align thousands of CMPs.

**Fig. 11:**
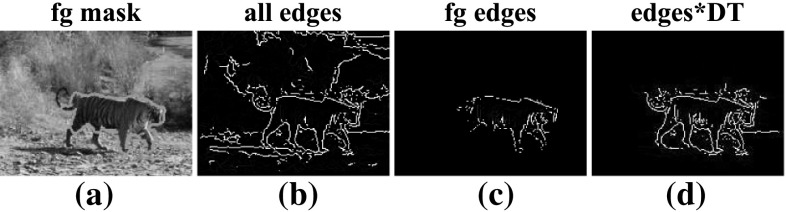
Edge extraction (Sect. 5.3.2). Using edges extracted from the entire image confuses the TTPS fitting due to background edge *points* (**b**). Using only *edges* on the foreground mask (**c**) loses useful edge *points* if the mask is inaccurate, e.g., the missing legs in **a**. We instead weigh the edge strength (**b**) by the distance transform (DT) with respect to the foreground mask. This is robust to errors in the mask, while pruning most background *edges* (**d**)

*Foreground edge points*. We extract edges using the edge detector (Dollar and Zitnick 2013) trained on the Berkeley segmentation dataset and benchmark (Martin et al. 2001). We remove clutter edges far from the object by multiplying the edge strength of each point with the distance transform (DT) of the image with respect to the foreground mask (i.e., the distance of each pixel to the closest point on the mask). We prune points scoring ${\le } 0.2.$ This removes most background edges, and is robust to cases where the mask does not cover the complete object (Fig. 11). To accelerate the TTPS fitting process, we further subsample the edge points to at most 1000 per frame.

## Related Work

### Learning from Videos

A few recent works exploit video as a source of training data for object class detectors (Leistner et al. 2011; Prest et al. 2012; Tang et al. 2013). They separate object instances from their background based on motion, thus reducing the need for manual bounding-box annotation. However, their use of video stops at segmentation. They make no attempt at modeling articulated motion or finding common motion patterns across videos. Ramanan et al. (2006) build a 2-D part-based model of an animal from one video. The model is a pictorial structure based on a 2-D kinematic chain of coarse rectangular segments. Their method operates strictly on individual videos and therefore cannot learn class models. It is tested on just three simple videos containing only the animal from a single constant viewpoint.

In the domain of action recognition, classification is typically formulated as a supervised problem (Schuldt et al. 2004; Kuehne et al. 2011; Soomro et al. 2012). Work on unsupervised motion analysis has largely been restricted to the problem of dynamic scene analysis (Kuettel et al. 2010; Hospedales et al. 2009; Mahadevan et al. 2010; Wang et al. 2009; Hu et al. 2006; Zhao and Medioni 2011). These works typically consider a fixed scene observed at a distance from a static camera; the goal is to model the behavior of agents (typically pedestrians and vehicles) and to detect anomalous events. Features typically consist of optical flow at each pixel (Hospedales et al. 2009; Kuettel et al. 2010; Wang et al. 2009) or single trajectories corresponding to tracked objects (Hu et al. 2006; Zhao and Medioni 2011).

Although many approaches do not easily transfer from the supervised to the unsupervised setting, a breakthrough from the action recognition literature that does is the concept of *dense trajectories*. The idea of generating trajectories for each object from large numbers of KLT interest points in order to model its articulation was simultaneously proposed by Matikainen et al. (2009) and Messing et al. (2009) for action recognition. These ideas were extended and refined in the work on tracklets (Raptis and Soatto 2010) and DTFs (Wang et al. 2011). IDTFs (Wang and Schmid 2013) currently provide state-of-the-art performance on video action recognition (Jiang et al. 2014).

### Representations Related to PoTs

In contrast to PoTs, most trajectory-based representations treat each trajectory in isolation (Wang et al. 2011; Wang and Schmid 2013; Messing et al. 2009; Matikainen et al. 2009; Raptis and Soatto 2010). Two exceptions are Jiang et al. (2012) and Narayan and Ramakrishnan (2014). Jiang et al. (2012) assign individual trajectories to a single codeword from a predefined codebook (as in DTF works Wang et al. 2011; Wang and Schmid 2013). However, the codewords from a PoTs are combined into a ‘codeword pair’ augmented by coarse information about the relative motion and average location of the two trajectories. Yet, this pairwise analysis is cursory: the selection of codewords is unchanged from the single-trajectory case, and the descriptor thus lacks the fine-grained information about the relative motion of the trajectories that PoTs provide. Narayan and Ramakrishnan (2014) model Granger causality between trajectory codewords. Their global descriptor only captures pairwise statistics of codewords over a fixed-length temporal interval. In contrast, a PoT groups two trajectories into a single local feature, with a descriptor encoding their spatiotemporal arrangement. Hence, PoTs can be used to find point correspondences between different videos (Fig. 14).

The few remaining methods that propose pairwise representations employ them in a very different context. Matikainen et al. (2010) use spatial and temporal features computed over pairs of sparse KLT trajectories to construct a two-level codebook for action classification. Dynamic-poselets (Wang et al. 2014) requires detailed manual annotations of human skeletal structure on training data to define a descriptor for pairs of connected joints. Raptis et al. (2012) consider pairwise interactions between clusters of trajectories, but their method also requires detailed manual annotation for each action. None of these approaches is suitable for unsupervised articulated motion discovery. If we consider pairwise representations in still images, Leordeanu et al. (2007) learned object classes by matching pairs of contour points from one image to pairs in another. Yang et al. (2010) computed statistics between local feature pairs for food recognition, again in still images.

### Unsupervised Behavior Discovery

To our knowledge, only Yang et al. (2013) considered the task of unsupervised behavior discovery, albeit from manually trimmed videos. Their method models human actions in terms of motion primitives discovered by clustering localized optical flow vectors, normalized with respect to the dominant translation of the object. In contrast, PoTs capture the complex relationships between the motion of two different object parts. Furthermore, we describe motion at a more informative temporal scale by using multi-frame trajectories instead of two-frame optical flow. We compare experimentally to Yang et al. (2013) on the KTH dataset (Schuldt et al. 2004) in Sect. 7.2.

### Spatial and Temporal Alignment

Most works on spatial alignment focus on aligning *still images* for a variety of applications: multi-view reconstruction (Seitz et al. 2006), image stitching (Brown and Lowe 2007), and object instance recognition (Ferrari et al. 2006; Lowe 2004). The traditional approach identifies candidate matches using a local appearance descriptor (e.g., SIFT Lowe 2004) with global geometric verification performed using RANSAC (Fischler and Bolles 1981; Chum and Matas 2008) or semi-local consistency checks (Schmid and Mohr 1996; Ferrari et al. 2006; Jegou et al. 2008). PatchMatch (Barnes et al. 2010) and SIFT Flow (Liu et al. 2008) generalize this notion to match patches between semantically similar scenes.

Our method differs from previous work on spatiotemporal *video sequence alignment* (Caspi and Irani 2000; Caspi et al. 2006; Ukrainitz and Irani 2006) in several ways. First, we find correspondences between different scenes, rather than between different views of the same scene (Caspi and Irani 2000; Caspi et al. 2006), potentially at different times (Evangelidis and Bauckhage 2013). While the method in Ukrainitz and Irani (2006) is able to align actions across different scenes by directly maximizing local space–time correlations, it cannot handle the large intra-class appearance variations and diverse camera motions present in our videos. As another key difference, all above approaches require temporally pre-segmented videos, i.e., they assume the two input videos show the same sequence of events in the same order and therefore can be aligned *in their entirety*. We instead operate with no available temporal segmentation, which is why we assume that only small portions of the videos can be aligned (the CMPs). Under stricter assumptions, our method can potentially align much longer sequences. Finally, these works have been evaluated only qualitatively on 5–10 pairs of sequences, whereas we provide extensive quantitative analysis (Sect. 7.4).

Several approaches focus on finding the optimal temporal alignment (i.e., frame-to-frame) between two or more video sequences (Tuytelaars and van Gool 2004; Wang et al. 2014; Douze et al. 2015; Dexter et al. 2009; Rao et al. 2003). Some of these works use a cost matrix to find the alignment (Wang et al. 2014; Dexter et al. 2009) similarly to our CMP candidate extraction (Sect. 5.1). Also this class of methods assumes that the input sequences can be aligned in their entirety, or at least have a significant temporal overlap.

In the context of action recognition, there has been work on matching spatiotemporal templates to actor silhouettes (Gorelick et al. 2007; Yilmaz and Shah 2005) or groupings of supervoxels (Ke et al. 2007). Our work is different because we map pixels from one unstructured video to another. The method in Jain et al. (2013) mines discriminative space–time patches and matches them across videos. It focuses on rough alignment using sparse matches (typically one patch per clip), whereas we seek a finer, non-rigid spatial alignment. Other works on sequence alignment focus on temporal rather than spatial alignment (Rao et al. 2003) or target a very specific application, such as aligning presentation slides to videos of the corresponding lecture (Fan et al. 2011).

A few methods use TPS for non-rigid point matching between still images (Chui and Rangarajan 2003), and to match shape models to images (Ferrari et al. 2010). TPS were initially developed as a general purpose smooth functional mapping for supervised learning (Wahba 1990). The computer graphics community recently proposed semi-automated video morphing using TPS (Liao et al. 2014). However, this method requires manual point correspondences as input, and it matches image brightness directly.

## Experiments

### Dataset

To evaluate our system, we assembled a new dataset of video shots for three highly articulated classes: tigers (500 shots), horses (100) and dogs (100). The horse and dog shots are primarily low-resolution footage filmed by amateurs (YouTube), while the tiger shots come from high-resolution National Geographic documentaries filmed by professionals. This enables quantitative analysis on a large scale in two very different settings.

We automatically partition each tiger video into shots by thresholding color histogram differences in consecutive frames (Kim and Kim 2009), and kept only shots showing at least one tiger. Horse and dog shots are sourced from the YouTube-objects dataset (Prest et al. 2012), where each shot contains at least one instance.

**Table 1:** Number of intervals recovered per behavior on tigers (top), horses (middle) and dogs (bottom)

Tiger partitions	Walk	Turn head	Sit down	Tilt head	Stand up	Drag	Wag tail	Walk back	Run	Turn	Jump	Raise paw	Open mouth	Close mouth	Blink	Slide leg	Drink	Chew	Lick	Climb	Roll	Scratch	Swim
Whole shots	259	24	5	17	5	4	1	5	16	3	9	0	4	0	0	1	7	4	6	1	3	1	3
Pauses	272	76	11	30	9	4	2	6	16	2	11	2	11	3	10	2	7	5	7	1	5	1	3
Pauses + period	**273**	**80**	**13**	**33**	**9**	**4**	**2**	**6**	**16**	**3**	**11**	**2**	**12**	**3**	**11**	**3**	**8**	**5**	**7**	**1**	**5**	**1**	**3**
Ground truth	289	148	27	77	24	4	4	10	23	18	20	6	40	28	39	13	12	7	19	1	5	2	3

Horse partitions	Walk	Turn head	Sit down	Tilt head	Stand up	Drag	Walk back	Gallop	Turn	Jump	Raise paw	Trot	Piaffe	Jump hurdles	Graze	Rodeo	Rolling						
Whole shots	16	2	1	5	1	3	0	30	2	1	0	17	3	6	1	4	1						
Pauses	16	2	1	7	1	3	0	31	3	1	0	20	3	6	1	4	1						
Pauses + period	**19**	**4**	**1**	**8**	**2**	**3**	**1**	**33**	**4**	**1**	**0**	**20**	**3**	**6**	**1**	**4**	**1**						
Ground truth	27	11	1	12	2	3	1	39	11	2	1	22	3	7	6	4	3						

Dog partitions	Walk	Turn head	Sit down	Tilt head	Stand up	Walk back	Run	Turn	Jump	Open mouth	Close mouth	Blink	Slide leg	Lick	Push skateboard								
Whole shots	25	4	0	1	0	0	10	0	4	0	0	0	0	0	13								
Pauses	29	5	0	1	0	0	12	2	5	0	0	0	0	0	16								
Pauses + period	**29**	**9**	**0**	**3**	**0**	**1**	**13**	**5**	**5**	**0**	**0**	**0**	**0**	**0**	**16**								
Ground truth	39	25	1	12	1	2	20	14	8	2	1	2	1	1	19								

Bold values indicate best performance

Pauses + periodicity consistently dominates others (Sect. 7.3)

We provide two levels of ground-truth annotations: behavior labels to evaluate PoTs (Sect. 7.2) and the behavior discovery stage (Sect. 7.3), and 2-D landmarks to evaluate the spatial alignment stage (Sect. 7.4). We publicly released this data at Del Pero et al. (2015b), where we also provide foreground masks for each shot computed using Papazoglou and Ferrari (2013).

*Behavior labels*. We annotated all the frames in the dataset (110,000) with the behavior displayed by the animal, choosing from the labels in Table 1. As animals move over time, often a shot contains more than one label. Therefore, we annotated each frame independently. When a frame shows multiple behaviors, we chose the one that appears at the larger scale (e.g., “walk” over “turn head”, “turn head” over “blink”). If several animals are visible in the same frame, we annotated the behavior of the one closest to the camera.

**Fig. 12:**
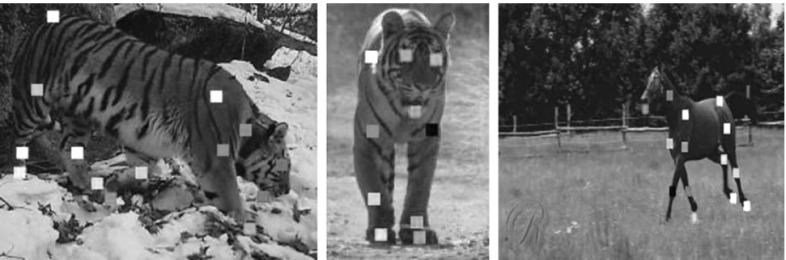
Examples of annotated landmarks. A total of 19 *points* are marked when visible in over 17,000 frames for two different classes (*horses* and *tigers*). Our evaluation measure uses the landmarks to evaluate the quality of a sequence alignments (Sect. 7.4)

*Landmarks*. We annotated the 2-D location of 19 landmarks (Fig. 12) in all the 16,000 frames of the horse class, and in 17,000 of the tiger class (Tiger_val, see below). For horses we annotated: eyes (2), neck (1), chin (1), hooves (4), hips (4) and knees (4). For tigers: eyes (2), neck (1), chin (1), ankles (4), feet (4) and knees (4). We did not annotate occluded landmarks. Unlike coarser annotations, such as bounding boxes, landmarks enable evaluating the alignment of objects with non-rigid parts with greater accuracy. Again, if several animals are visible in the same frame, we annotated the one closest to the camera.

*Tiger subsets*. We now define three different subsets of the tiger shots, which we use throughout the experiments. *Tiger_all* denotes all tiger shots. *Tiger_val* contains 100 randomly selected shots used to set the parameters of the methods we test. *Tiger_fg* contains 100 manually selected shots in which the method of Papazoglou and Ferrari (2013) produced accurate foreground masks (with no overlap with Tiger_val). We use Tiger_fg to assess how sensitive the methods are to the accuracy of the foreground masks. All other subsets are instead representative of the average performance of Papazoglou and Ferrari (2013) (which is accurate on ${\sim }55\,\%$ of the cases).

### Evaluation of PoTs

We first evaluate PoTs (Sect. 3) in a simplified scenario where we cluster intervals for which the correct single-behavior partitioning is given, i.e., we partition shots at frames where the ground-truth behavior label changes. This allows us to evaluate the PoT representation separately from our method for automatic behavior discovery, which does the partitioning automatically (Sect. 7.3).

#### Evaluation Protocol

We compare PoTs to the state-of-the-art IDTFs (Wang and Schmid 2013). IDTFs combine four different feature channels aligned with dense trajectories: TS, HOGs, histogram of optical flow (HOF), and MBH. TS is the channel most related to PoTs, as it encodes the displacement of an individual trajectory across consecutive frames. HOG is the only component based on appearance and not on motion. We also compare against a version of IDTFs where only trajectories on the foreground masks are used, which we call fg-IDTFs. We use the same point tracker (Wang and Schmid 2013) to extract both IDTFs and PoTs. For PoTs, we do not remove trajectories that are static or are caused by the motion of the camera. Removing these trajectories improves the performances of IDTFs (Wang and Schmid 2013), but in our case they are useful as potential anchors.

We adopt two criteria commonly used for evaluating clustering methods: *purity* and *adjusted rand index* (ARI; Rand 1971). Purity is the number of items correctly clustered divided by the total number of items (an item is correctly clustered if its label coincides with the most frequent label in its cluster). While purity is easy to interpret, it only penalizes assigning two items with different labels to the same cluster. The ARI instead also penalizes putting two items with the same label in different clusters. Further, it is adjusted such that a random clustering will score close to 0. It is considered a better way to evaluate clustering methods by the statistics community (Hubert and Arabie 1985; Santos and Embrechts 2009).

*Parameter setting*. We use Tiger_val to set the PoT selection threshold $\theta _{P}$ (Sect. 3.2) and the PoT codebook size *V* (Sect. 4.2) using coarse grid search. As objective function, we used the ARI achieved by our method when the number of clusters is equal to the true number of behaviors. We used interval [0.05, 0.35] with a step of 0.05 for $\theta _{P},$ and [800, 8000] with a step of 800 for *V*. Grid search selects $\theta _{P}=0.15,\,V=800$ and we use these values in all experiments on all classes. In practice, performance is very similar for a wide range of parameters: $0.1 \le \theta _{P} \le 0.25$ and $800 \le V \le 1600$. We tuned the IDTFs codebook size analogously and found that 4000 codewords work best. Interestingly, the same value is chosen by Wang and Schmid (2013) on completely different data.

**Fig. 13:**
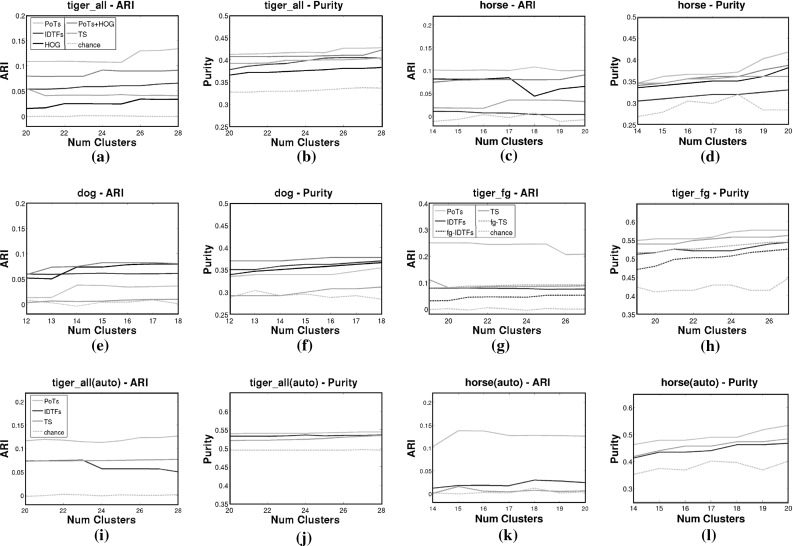
Results of clustering intervals using different descriptors (Sect. 7.2.2), evaluated on adjusted rand index (ARI) and purity (Sect. 7.2.1). PoTs result in better clusters than IDTFs (Wang and Schmid 2013) on tigers and horses (**a**–**d**). Adding appearance (PoTs + HOG) is detrimental on these two classes, but improves performance on dogs (**e**, **f**). IDTFs perform well for dogs, primarily due to the contribution of the HOG channel: compare the full descriptor (*blue*), with the HOG channel only (*black*) and the trajectory shape channel TS (*magenta*). For all classes, PoTs + HOG performs better than IDTFs. The gap between IDTFs and PoTs increases on tiger_fg, where we ensured the segmentation is accurate (**g**, **h**). Here, PoTs also outperform IDTFs extracted on the foreground mask only (fg-IDTFs). PoTs also generate higher-quality clusters than the other methods when we cluster automatically partitioned intervals (**i**–**l**, Sect. 7.3) (Color figure online)

#### Results

We compare clustering using BoWs of PoTs to using BoWs of IDTFs in Fig. 13a–h. As the true number of clusters is usually not known a priori, each plot shows performance as a function of the number of clusters. The mid value on the horizontal axis corresponds to the true number of behaviors (23 for tigers, 17 for horses, 15 for dogs).

For tigers and horses, the clusters found using PoTs are better in both purity and ARI, compared to using IDTFs (Fig. 13a–d). Consider now the individual IDTFs channels. On tigers, the HOG channel performs poorly, and adding it to PoTs (PoTs + HOG) performs worse than PoTs alone. Appearance is in general not suitable for distinguishing between fine-grained behaviors. It is particularly misleading when different object instances have similar color and texture (like tigers). The HOF and MBH channels of IDTF perform poorly on their own and are not shown in the plot.

The gain over IDTFs is larger on Tiger_fg (g, h), where PoTs benefit from the accurate foreground masks. Here, PoTs also outperform fg-IDTFs, showing that the power of our representation resides in the principled use of PoTs, not just in exploiting foreground masks to remove background trajectories. Moreover, all other results (a–f) show that PoTs can also cope with imperfect masks.

For the dog class, IDTFs perform better than PoTs (Fig. 13e, f). However, HOG is doing most of the work in this case. The dog shots come from only eight different videos, each showing one particular dog performing one–two behaviors in the same scene. Hence, HOG performs well by trivially clustering together intervals from the same video. When we equip PoTs with HOG, they outperform the complete IDTFs. Additionally, if we consider trajectory motion alone PoTs outperform TS, further confirming that PoTs are a more suitable representation for articulated motion.

Results on tigers and horses showed that adding appearance features can be detrimental, since there is little correlation between a behavior and the appearance of the animal and/or the background. This is not the case for the dog class, where the shots come from only 8 different scenes, compared to more than 50 for horses, and several hundreds for tigers. However, it shows that PoTs and appearance features are complementary: when appearance should be beneficial, we see the expected performance boost by adding this additional information. This is potentially useful for traditional action recognition tasks (Soomro et al. 2012; Karpathy et al. 2014), where many activities strongly correlate with the background and the apparel involved (e.g., diving can be recognized from the appearance of swimsuits, or a diving board with a pool below). Last, we note that we use the same PoT parameters on all datasets (set on Tiger_val, Sect. 7.2.1), showing that our representation generalizes across classes.

**Table 2:** Interval uniformity for different partitioning methods

	Whole shots	Pauses	Pauses + periods	Ground truth
Tiger # intervals	480	719	**885**	1026
Tiger uniformity	0.78	0.85	**0.87**	1
Horse # intervals	96	117	**184**	194
Horse uniformity	0.82	0.83	**0.89**	1
Dog # intervals	80	115	**219**	260
Dog uniformity	0.72	0.80	**0.88**	1

Bold values indicate best performance

Pauses + periods consistently outperforms alternatives (Sect. 7.3)

**Fig. 14:**
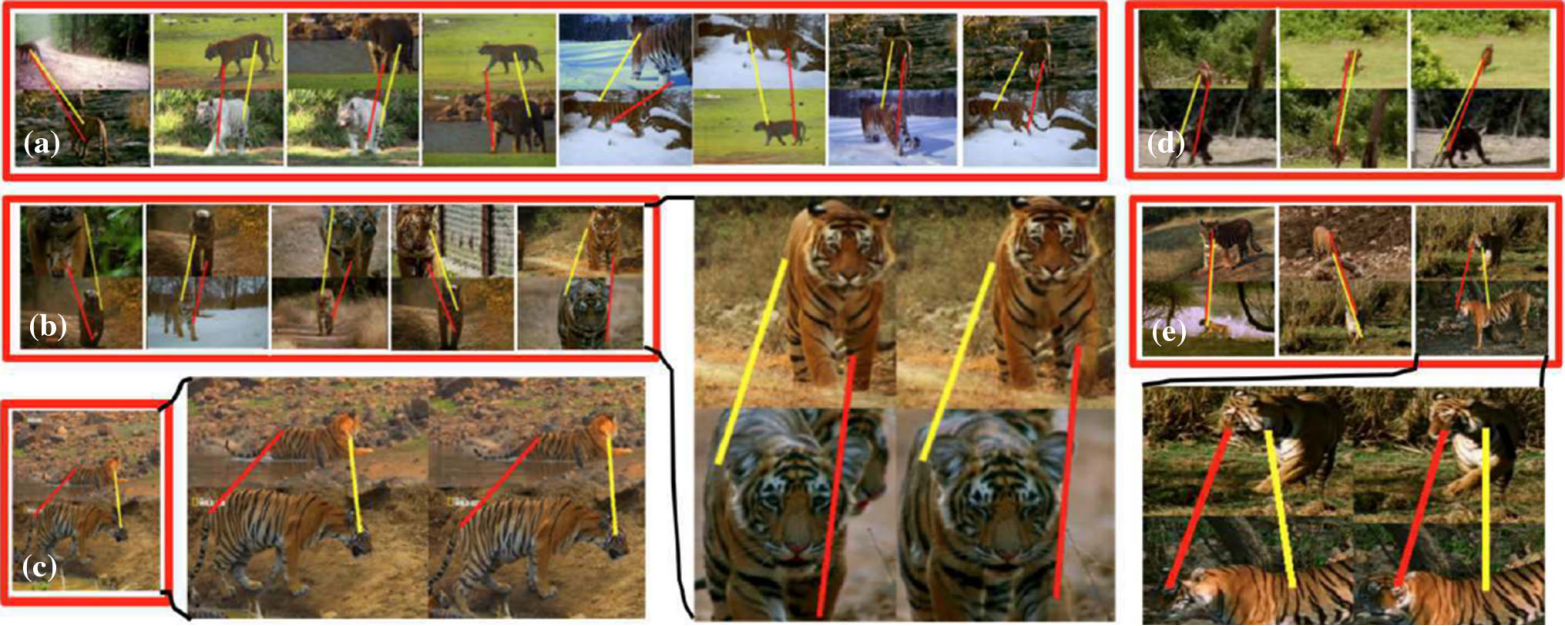
Behaviors discovered by clustering consistent motion patterns (Sect. 7.3). Each *red rectangle* displays a few pairs of intervals from one cluster, on which we connect the anchors (*yellow*) and swings (*red*) of two individual PoTs that are close in descriptor space. The enlarged version show how the connected PoTs evolve through time, and give a snapshot of one representative motion pattern for each cluster. The behaviors shown are: two different ways of walking (**a**, **b**), sitting down (**c**), running (**d**), and turning *head* (**e**). Video showing behavior clusters for all classes are available on our website (Del Pero et al. 2015b) (Color figure online)

*Comparison to motion primitives* (Yang et al. 2013) last, we compare to the method of Yang et al. (2013), which is based on motion primitives (Sect. 6.3). Since they did not release their method, we compare to the results they report on the KTH dataset (Schuldt et al. 2004) in their setting. The KTH dataset contains 100 shots for each of 6 different human actions (e.g., walking, hand clapping). As before, we cluster all shots using the PoT representation: for the true number of clusters (6), we achieve 59 % purity, compared to their 38 % (Fig. 9 in Yang et al. 2013). For this experiment, we incorporated an R-CNN person detector (Girshick et al. 2014) into Papazoglou and Ferrari (2013) to better segment the actors.

### Evaluation of Behavior Discovery

We first evaluate our method for partitioning shots into single-behavior intervals (Sect. 4.1). Let the uniformity of an interval be the number of frames with the most frequent label in it, divided by the total number of frames. The combination of pauses and periodicity partitioning improves the baseline average interval uniformity of the original, unpartitioned shots (Table 2). This is very promising, since the average uniformity is near $90\,\%,$ and the number of intervals found approaches the ground-truth number. In Table 1 we report the number of single-behavior intervals found by each method, grouped by behavior. We only increase the count for intervals from different shots, otherwise we could approach the ground-truth number by simply partitioning one continuous behavior into smaller and smaller pieces (e.g., if our method returns three intervals from the same shot whose ground-truth label is “walking”, we increase the count for “walking” in Table 2 only by one). We chose this counting method because our ultimate goal is to find instances of the same behavior performed by different object instances. Clustering whole shots would lose many behaviors, and only a few dominant ones such as walking would emerge. Our method instead finds intervals for almost all behavior types.

Last, we report purity and ARI for the clusters of partitioned intervals (Fig. 13i–l). As ground-truth label for a partitioned interval, we use the ground-truth label of the majority of the frames in it. PoTs outperform IDTFs on tigers and horses also in this setting. To make this comparison fair, we evaluate IDTFs and PoTs after using the same partitioning method (pauses + periodicity). We show a few qualitative examples of the discovered behavior clusters in Fig. 14.

### Evaluation of Sequence Alignment

The input of this experiment are the clusters of intervals discovered by our method (step 3 in Fig. 2). We set the number of clusters to be a fourth of the number of intervals in step 2. With this settings, the purity of the discovered clusters is above 0.7 (CMP extraction in step 4 benefits from having reasonably pure clusters as input). For the tiger class we only cluster the intervals in Tiger_val, since this is the only subset of the tiger class with landmark annotations (we use all intervals for horses).

We now introduce an alignment error measure (Sect. 7.4.1), which we use to evaluate CMP extraction (Sect. 7.4.2) and alignment (Sect. 7.4.3).

#### Alignment Error

We evaluate the mapping found between the two sequences in a CMP as follows. For each frame, we map each landmark in the first sequence onto the second and compute the Euclidean distance to its ground-truth location. The error for the landmark is the average between this distance and the reverse (i.e., when we map the landmark from the second sequence into the first). We normalize the error by the scale of the object, defined as the maximum distance between any two landmarks in the frame. The overall alignment error is the average error of all visible landmarks over all frames.

**Fig. 15:**
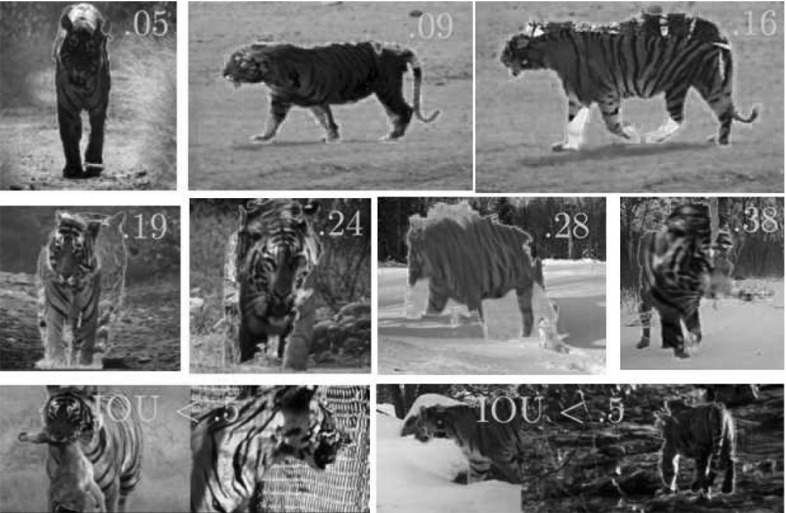
Alignment error. We use the ground-truth landmarks to measure the alignment error of the mappings estimated by our method (Sect. 7.4.1). As the error increases, the quality of the alignment clearly degrades. Around 0.18 the alignments contain some slight mistakes (e.g., the slightly misaligned *legs* in the *top right image*), but are typically acceptable. We consider an alignment incorrect when the error is above 0.18, and also when the IOU of the visible landmarks in the aligned pair is below 0.5 (*bottom row*)

After visual inspection of many sampled alignments (Fig. 15), we found that 0.18 was a reasonable threshold for separating acceptable alignments from those with noticeable errors. We count an alignment as correct if the error is below this threshold and if the intersection-over-union (IOU) of the two sets of visible landmarks in the sequences is above 0.5.^3^ This prevents rewarding accidental alignments of a few landmarks (bottom row of Fig. 15).

#### Results on CMP Extraction

First, we evaluate our method for CMP extraction in isolation (Sect. 5.1). Given a CMP, we fit a homography to correspondences between the ground-truth landmarks, and check if it is correct based on the alignment error above. This indicates that it is possible to align the CMP (we call it *alignable*). Computing (5) using both PoTs and MBH returns roughly 3000 CMP on tigers, of which $51\,\%$ are alignable ($43\,\%$ if we use only PoTs). As a baseline, we extract CMPs directly from the input shots: we select the starting frames of the two sequences in a CMP by sampling from a uniform distribution over all input frames (i.e., without steps 2 and 3 in Fig. 2). The percentage of alignable CMPs produced by this baseline is only $19\,\%.$ Results are similar on horses: our method delivers $49\,\%$ alignable CMPs ($47\,\%$ using only PoTs), versus $26\,\%$ by the baseline.

#### Results on Spatial Alignment

We now evaluate our methods for sequence alignment (Sects. 5.2, 5.3). For each, we generate a precision–recall curve as follows. Let *n* be the total number of CMPs returned by the method, *c* the number of correctly aligned CMPs, and *a* the total number of alignable CMPs (Sect. 7.4.2). Recall is *c* / *a*,  and precision is *c* / *n*. Different operating points on the precision–recall curve are obtained by varying the maximum percentage of outliers allowed when fitting a homography.

*Baselines* we compare our method against SIFT Flow (Liu et al. 2008). We use SIFT Flow to align each pair of frames from the two CMP sequences independently. We help the SIFT Flow algorithm by matching only the bounding boxes of the foreground masks, after rescaling them to be the same size. Without these two steps, the algorithm fails on most CMPs.

We also compare to fitting a homography to SIFT matches between the two sequences. We use only keypoints on the foreground mask, and preserve temporal order by matching only keypoints in corresponding frames. We tested this method alone (SIFT), and by adding spatial regularization with the foreground masks (SIFT + FG, as in Sect. 5.2.3). Finally, we consider a simple baseline that fits a homography to the bounding boxes of the foreground masks alone (FG).

**Fig. 16:**
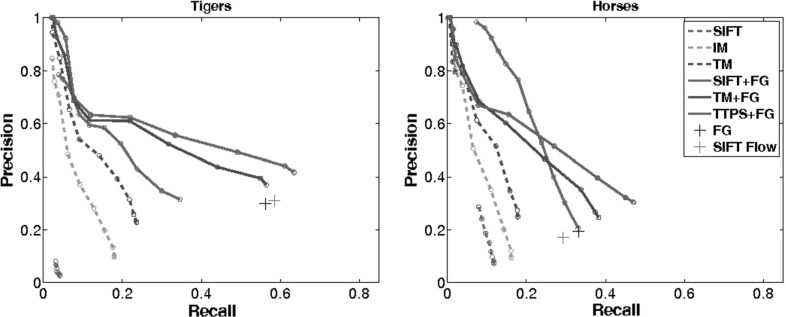
Evaluation of sequence alignment. We separately evaluate our method on two classes, horses and tigers (Sect. 7.4.3). With no regularization, trajectory methods are superior to SIFT on both classes, with TM performing better than IM. Adding regularization using the foreground masks (+FG) improves the performance of both TM and SIFT (compare the *dashed* to the *solid curves*). TTPS clearly outperform all trajectory methods, as well as SIFT Flow and the FG baseline (Sect. 7.4.3)

**Fig. 17:**
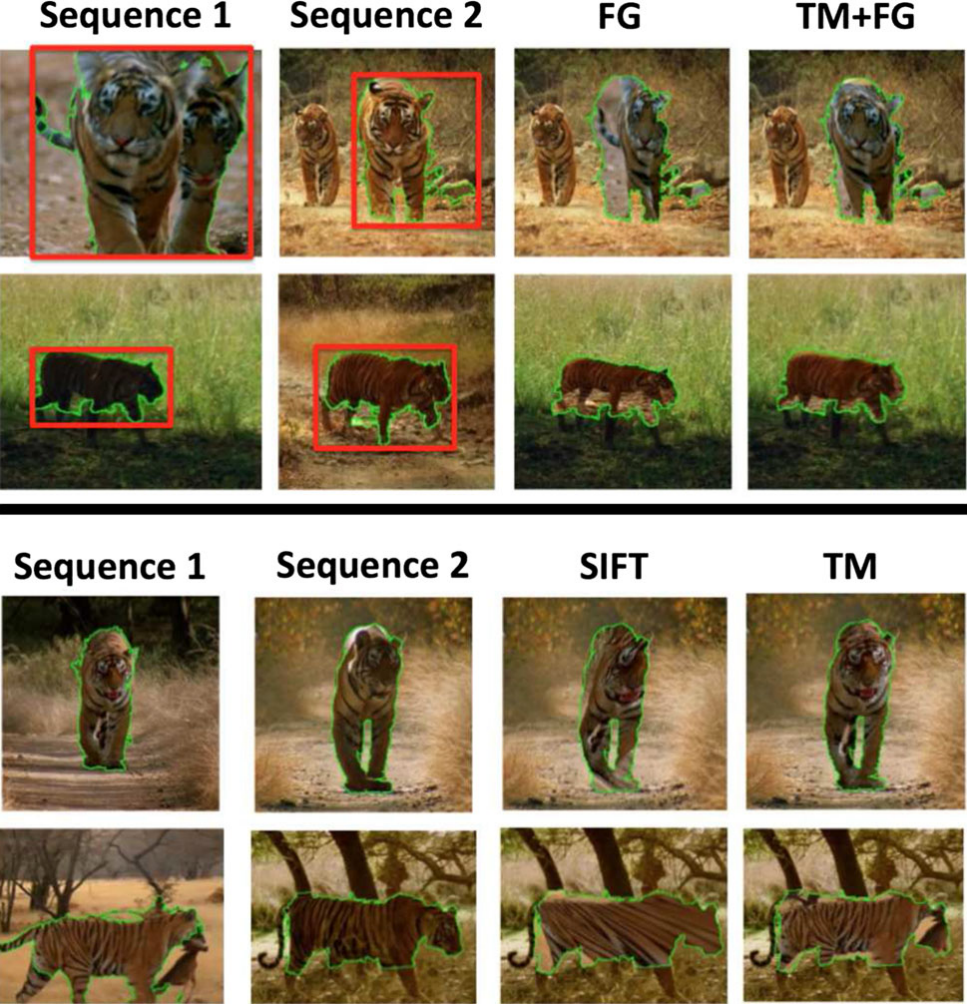
*Top two rows:* estimating the homography from the foreground masks alone (FG) fails when the bounding *boxes* are not tight around the objects (*first–second columns*). Adding trajectories (TM + FG) is more accurate (Sect. 5.2.3). *Bottom two rows:* the striped texture of *tigers* often confuses estimating the homography from SIFT keypoint matches (*third column*). On this class, using trajectories (TM) often performs better (Sect. 7.4.3)

We report results in Fig. 16. Among the homography-based methods (Sect. 5.2), those using trajectory correspondences (TM, IM, Sect. 5.2.2) are superior to using SIFT on both classes, with TM outperforming IM. Adding spatial regularization with the foreground masks (+FG) improves the performance of both TM and SIFT. SIFT performs poorly on tigers, since the striped texture confuses matching SIFT keypoints (Fig. 17, bottom). Methods using trajectories work somewhat better on tigers than horses due to the poorer quality of YouTube video (e.g., low resolution, shaky camera, abrupt pans). As a consequence, TM + FG clearly outperforms SIFT + FG on tigers, but it is somewhat worse on horses.

The TTPS model (TTPS + FG, Sect. 5.3) significantly improves upon its initialization (TM + FG) on both classes. On tigers, it is the best method overall, as its performance curve is above all others for the entire range. On horses, the SIFT + FG and TTPS + FG curves intersect. However, TTPS + FG achieves a higher average precision (i.e., the area under the curve): 0.265 versus. 0.235.

**Fig. 18:**
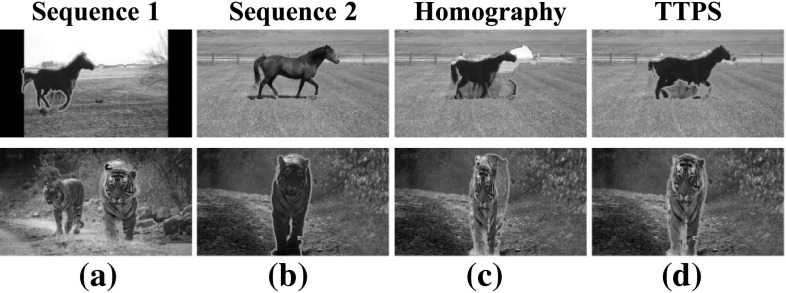
Given two input sequences of articulated objects (**a**, **b**), TTPS often provide a more accurate alignment (**d**) than homographies (**c**, Sect. 7.4.3)

The SIFT Flow software (Liu et al. 2008) does not produce scores comparable across CMPs, so we cannot produce a full precision–recall curve. At the level of recall of SIFT Flow, TTPS + FG achieves +0.2 higher precision on tigers, and +0.3 on horses. We also note that TM and TM + FG are closely related to the method for fitting homographies to trajectories in Caspi et al. (2006). Although TM + FG extends (Caspi et al. 2006) in several ways (automatic CMP extraction, modified TS descriptor, regularization with foreground masks), it is still inferior to TTPS + FG. Last, TTPS + FG also achieves a significantly higher precision than the FG baseline. This shows that our method is robust to errors in the foreground masks (Fig. 17, top). Head-to-head qualitative results show that TTPS + FG alignments typically look more accurate than the other methods (Fig. 18). A video with many examples is available on our website (Del Pero et al. 2015b).

For the tiger class, out of all CMPs returned by TTPS + FG (rightmost point on the curve), 1000 of them are correctly aligned (i.e., 10,000 frames). The precision at this point is 0.5, i.e., half of the returned CMPs are correctly aligned. For the horse class, TTPS + FG returns 800 correctly aligned CMPs, with precision 0.35.

**Table 3:** Run-time of the main steps of our method (Sect. 7.5)

Steps	Run-time (s)
Optical flow (Brox and Malik 2011, per frame)	1.5
Foreground mask (per frame)	0.5
Dense trajectory extraction (per frame)	0.4
PoT extraction (per frame)	0.1
Homography alignment (per CMP)	5
TTPS alignment (per CMP)	44

### Runtime

We report the run-time of the main steps of our method in Table 3, including pre-processing. We measured run-time on a Dell server with a 1.6 GHz CPU and 16 GB RAM. The PoT extraction time is negligible compared to the pre-processing steps (optical flow, foreground mask and dense trajectory extraction). We note that large video collections can be processed efficiently on a computer cluster, since each input shot (or CMP for the alignment) can be processed independently.

### Analysis of Failures and Limitations

**Fig. 19:**
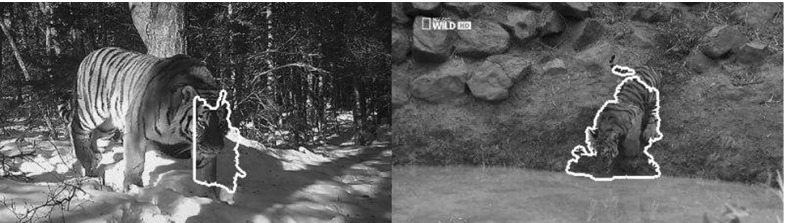
Failures due to inaccurate foreground mask. Our system is robust to inaccuracies in the foreground masks (Sect. 4.2 and Fig. 11), but cannot recover when the object is almost completely missed (*left*). Here the walking *tiger* (*left*) was clustered with the *tiger* sitting down (*right*) during behavior discovery (Sect. 4.2). This in turn breaks the alignment stage, as these two *tigers* cannot be aligned via homography or TTPS (Sect. 5). We estimated by visual inspection that complete failures in the masks happen in roughly $15\,\%$ of the input shots (Sect. 7.6)

*Inaccurate foreground masks*. Our system is robust to small to medium inaccuracies in the foreground masks, such as missing part of the object or including some of the background (see Sect. 4.2 and Fig. 11). However, we cannot cope with catastrophic failures, for example when the object is completely missed. In these cases the PoT extraction is not reliable, which results in assigning such shots to the wrong behavior cluster (Fig. 19), which in turn produces wrong alignments in the following step of our system. However, these problematic cases are not frequent (about $15\,\%$ of the input shots). Moreover, we noticed that hierarchical clustering often puts such an item in a singleton cluster, which mitigates the problem. Inaccuracies in the masks can potentially be detected and fixed by co-segmenting all the intervals in a behavior cluster, while enforcing consistent appearance and shape across all their foreground masks.

*Scale and viewpoint invariance*. The PoT descriptor is invariant to scale (Sect. 3.1). In general, smaller objects will generate fewer trajectories (hence fewer PoTs), but this is not a problem since we aggregate the PoTs into a normalized BOW histogram (Sect. 4.2). Our results show that our method clusters together objects at a very different scale (e.g., Fig. 14b). Only cases where the object is very small are problematic (${<}50 \times 50$ pixels). PoTs are also robust to moderate viewpoint and pose variations. However, they cannot cope with drastic viewpoint difference, e.g., a video of a tiger walking frontally and one walking to the right. Establishing correspondences between clusters showing the same behavior under widely different viewpoints is an interesting research direction.

*Camera motion*. The PoT descriptor can cope with camera panning, and other moderate camera motions (Sect. 3.1). The foreground masks also help in the presence of panning, since the motion of rigid regions of the object and the background would be indistinguishable in this case. However, fast zooming can be problematic.

*Extensions to multiple classes*. The main goal of our system is to organize a collection of videos of the same class. However, extensions to multiple classes are possible. In the case of related classes (e.g., quadrupeds), similar behaviors of different classes might be grouped together, and additional cues might be needed to separate them.

## Discussion

We introduced a weakly supervised system that discovers the behaviors of an articulated object class from unconstrained video, while also spatially aligning several instances of each behavior. We emphasize that the only supervision needed is a single label per video, indicating which class it contains.

The entire system is bottom-up and needs not relate to the kinematic structure of an object class. We showed that the behavior discovery and the alignment process apply to different classes, by leveraging the recurring motion patterns of a particular class, rather than being limited to pre-defined relationships.

This was enabled by our PoT descriptor, which proves very effective for modeling the motion of articulated objects. Thanks to the use of PoTs, PoTs outperform alternative motion descriptors (e.g., TS) on behavior discovery. While being appearance-free, on horses and tigers PoTs also outperform all tested alternatives that included appearance information (e.g., IDTFs). When augmented with appearance descriptors, PoTs also outperforms competitors on the dog class. In terms of spatial alignment, we have shown that our technique produces more accurate alignments than relevant alternatives such as SIFT Flow and SIFT matching.

Thanks to the principled use of motion, we discovered behaviors and recovered alignments across instances exhibiting significant appearance variations (orange and white tigers, cubs and adults, etc.). Establishing such correspondences across different object instances can be very useful to learn class-level models of behavior and/or appearance. Our method recovers them automatically from unconstrained Internet video, and can be a platform for replacing the tedious and expensive manual annotations normally needed when learning from video.

## Footnotes

1 Since the PoT descriptor is heterogenous (Sect. 3.1), we ran preliminary experiments on held-out data to weigh the relative importance of $\theta$ and the displacement vectors (analogously to the way we set the other parameters, Sect. 7.2.1). We found the optimal weight is 1.

2 Consider a simple example with $T=2,$ where we extract 10 points at $t=1$ and 20 at $t=2{\text {:}}\,\mathcal {U}^1$ and $\mathcal {U}^2$ contain 30 points; the first 10 in $\mathcal {U}^1$ are the point extracted at $t=1,$ the next 20 those extracted at $t=2$ and propagated to $t=1$ with the backward flow; the first 10 in $\mathcal {U}^2$ are the points extracted at $t=1$ propagated to $t=2$ with forward flow, the next 20 those extracted at $t=2.$

3 If $\mathcal {L}_{1}$ is the set of landmarks visible in the first sequence in a CMP, and $\mathcal {L}_{2}$ those in the second, IOU$(\mathcal {L}_{1},\,\mathcal {L}_{2})=|\mathcal {L}_{1} \cap \mathcal {L}_{2}|/|\mathcal {L}_{1} \cup \mathcal {L}_{2}|.$ For example, if $\mathcal {L}_{1}$={left_eye,right_eye,neck} and $\mathcal {L}_{2}$={front_right_knee, right_shoulder, neck}, IOU=1 / 5.
